# Pulmonary Hypertension Remodels the Genomic Fabrics of Major Functional Pathways

**DOI:** 10.3390/genes11020126

**Published:** 2020-01-23

**Authors:** Rajamma Mathew, Jing Huang, Sanda Iacobas, Dumitru A. Iacobas

**Affiliations:** 1 Department of Pediatrics, New York Medical College, Valhalla, NY 10595, USA; rmathew@nymc.edu (R.M.); jinghuang100@gmail.com (J.H.); 2 Department of Physiology, New York Medical College, Valhalla, NY 10595, USA; 3 Department of Pathology, New York Medical College, Valhalla, NY 10595, USA; 4 Personalized Genomics Laboratory, Center for Computational Systems Biology, Roy G Perry College of Engineering, Prairie View A&M University, Prairie View, TX 77446, USA

**Keywords:** aerobic glycolysis, caveolin1, hypoxia, monocrotaline, oxidative phosphorylation, RhoA

## Abstract

Pulmonary hypertension (PH) is a serious disorder with high morbidity and mortality rate. We analyzed the right-ventricular systolic pressure (RVSP), right-ventricular hypertrophy (RVH), lung histology, and transcriptomes of six-week-old male rats with PH induced by (1) hypoxia (HO), (2) administration of monocrotaline (CM), or (3) administration of monocrotaline and exposure to hypoxia (HM). The results in PH rats were compared to those in control rats (CO). After four weeks exposure, increased RVSP and RVH, pulmonary arterial wall thickening, and alteration of the lung transcriptome were observed in all PH groups. The HM group exhibited the largest alterations, as well as neointimal lesions and obliteration of the lumen in small arteries. We found that PH increased the expression of caveolin1, matrix metallopeptidase 2, and numerous inflammatory and cell proliferation genes. The cell cycle, vascular smooth muscle contraction, and oxidative phosphorylation pathways, as well as their interplay, were largely perturbed. Our results also suggest that the upregulated *Rhoa* (Ras homolog family member A) mediates its action through expression coordination with several ATPases. The upregulation of antioxidant genes and the extensive mitochondrial damage observed, especially in the HM group, indicate metabolic shift toward aerobic glycolysis.

## 1. Introduction

Pulmonary hypertension (PH), characterized by increasing pulmonary artery pressure and vascular resistance [1], is a serious complication of a number of unrelated cardiopulmonary, inflammatory, and autoimmune diseases, as well as of drug toxicity. PH is characterized by endothelial dysfunction, enhanced vasoconstrictor reactivity, activation of proliferative and anti-apoptotic pathways, vascular remodeling, elevated pulmonary artery pressure, and right-ventricular hypertrophy (RVH), leading to right-ventricular failure and premature death.

Recently updated PH classification maintains the original five groups with some modification [2]. The term pulmonary arterial hypertension (PAH) is assigned to Group1. It includes idiopathic (IPAH) and heritable PAH (HPAH), PAH associated with congenital heart diseases, drug toxicity, connective tissue disorders, portal hypertension, human immunodeficiency virus (HIV) infection, and schistosomiasis. The average survival time in patients with PAH without treatment was reported in 1991 to be around 2.8 years [3]. Despite the improvement in quality of life, the current therapy fails to reverse or halt the progression of the vascular disease [4]. Progressive pulmonary vascular changes lead to neointimal lesions resulting in irreversibility of the PH [5]. The five-year survival rate remains poor in patients with IPAH, HPAH, and PAH associated with anorexigenic drug treatment [6,7]. PAH diagnosis is often delayed because of the vague symptoms. By the time the diagnosis is made, most of the patients have significant pathological changes in the pulmonary vasculature, which poses a serious challenge to therapeutic measures.

Mutations of genes *BMPR2* (bone morphogenetic protein receptor type 2) [8], *ACVRL1* (activin A receptor like type 1) [9], *ENG* (endoglin) [10], SMAD9 (SMAD family member 9) [11], *CAV1* (caveolin 1) [12], and *KCNK3* (potassium two-pore domain channel subfamily K member 3) [13] are well-documented causes of PAH.

About 20% of people with *BMPR2* mutation develop PH, indicating that a second hit is necessary, which could be mutations in additional transcription factors such as EIF2AK4 [14] or SMAD9 [15]. Importantly, patients with mutation and PAH tend to be younger [16]. In addition, 42% females who harbor BMPR2 mutation develop PAH compared with 14% males. This difference is attributed to the protective effect of Y-chromosome specific transcription factor SRY (sex-determined transcription region Y), which positively regulates BMPR2 [17].

Monocrotaline (MCT)- and hypoxia-induced PH are well-established animal models. MCT is a pyrrolizidine compound obtained from seeds and leaves of *Crotalaria spectabilis*. Following a single sc injection, MCT is converted in the liver to dehydroMCT, which, during the first passage through the lungs, causes pulmonary endothelial damage. By 72 h, the compound is completely excreted. Within one week, endothelial disruption and loss of endothelial membrane proteins such as Cav1, PECAM, and Tie2 occur. By two weeks post MCT, PH is observed [18].

Loss of Bmpr2 was observed in the MCT and hypoxia models of PH [19]. *BMPR2* gene therapy fails to inhibit MCT-induced PH; however, administration of GDF2 (growth differentiation factor 2, also known as BMP9) was shown to have preventive and reversal effect on experimental PH [20,21]. *BMPR2* is predominantly expressed in endothelial cells, and a part of it co-localizes with CAV1 [22], while the loss of BMPR2 increases the susceptibility to DNA damage [23]. The increased expression of *SMURF1* (SMAD-specific E3 ubiquitin protein ligase 1) that induces lysosomal and proteosomal degradation of BMPR2 was shown to occur in patients with PAH, as well as in hypoxia- and MCT-induced PH. Furthermore, Smurf1 deletion protects mice from PAH [19,24]. Robust expression of *Nos3* (nitric oxide synthase 3) was reported in plexiform lesions in PAH [25].

The importance of CAV1 in PH is supported by recent observations that patients with *CAV1* mutation develop PAH [12]. In addition, *Cav1* knockout mice develop PH which can be reversed by reconstituting endothelial Cav1 [26,27]. Extensive loss of endothelial CAV1 accompanied by enhanced expression of *CAV1* in vascular smooth muscle cells (VSMC) was reported in IPAH, in HPAH, in PAH associated with congenital heart defect and drug toxicity [22,28,29,30,31], and in infants with bronchopulmonary dysplasia and evidence of inflammation [29]. Furthermore, pulmonary artery smooth muscle cells isolated from patients with PH revealed enhanced expression of *CAV1*, increased capacitative Ca^2+^ entry, and increased DNA synthesis, which could be blocked by *CAV1* small interfering RNA (siRNA) [28]. Inflammation was shown to play a significant role in PH, and CAV1 modulates inflammatory response. Furthermore, *Cav1* knockout mice have increased levels of circulating pro-inflammatory cytokines [32]. In the experimental models of PH, loss of Cav1 was shown to be associated with increased levels and bioactivity of Il6, and activation of *Stat3* and *Nfkb1*. Rescue of Cav1 results in the attenuation of PH [32,33].

We previously showed that exposing the MCT-injected rats to hypoxia accelerates the PH disease process. By four weeks, there is extensive endothelial Cav1 loss, accompanied by enhanced expression of Cav1 in VSMC of a large number of arteries, resulting in near normalization of total Cav1 protein expression in the lungs. Several of the arteries with enhanced expression of Cav1 displayed neointima formation. Moreover, lung *Nos3* expression in this group was near normal levels unlike the group of MCT-injected rats exposed to normal atmosphere, where the lung Nos3 level was low. Importantly, lung sections from IPAH and HPAH patients revealed a similar sequence of events: (1) loss of endothelial CAV1 and endothelial cell disruption, (2) enhanced expression of CAV1 in VSMC, and (3) presence of neointima only in arteries that displayed enhanced expression of CAV1 in VSMC [30]. In vitro studies with pulmonary artery endothelial cells from IPAH patients have CAV1 degradation owing to sustained NOS3 and SRC signaling [34]. Furthermore, loss of CAV1 in endothelial cells was shown to cause mitochondrial stress, as well as increase oxidative stress and metabolic switch [35].

In this article, we present our observations on the four-week weight gain, right-ventricular systolic pressure (RVSP), right-ventricle hypertrophy (RVH), pulmonary vascular histology, and lung transcriptomes of three rat models of PH with respect to the control group. 

## 2. Materials and Methods 

### 2.1. Animals

The six-week-old male Sprague-Dawley rats (150–175g) were obtained from Charles River Wilmington, MA. Rats were allowed to acclimatize in the animal facility for five days and had free access to laboratory chow and water. The rats were kept in the animal facility maintained at 22 °C and 12 h each of light and dark cycle. The experiments respected the guiding principles for the use and care of laboratory animals of the American Physiological Society and the National Institutes of Health. The Institutional Animal Care and Use Committee at New York Medical College approved the protocol “Mechanism of neointima formation in pulmonary hypertension” (approval # 4-1-0113/2014, P.I., R.M). Rats were divided into four groups (*n* = 8/group). Gr1, control rats (CO), were maintained in room air. Gr2 (CM) rats received a single subcutaneous injection of monocrotaline (MCT 40 mg/kg) and were kept in room air. Gr3 (HO) rats were subjected to hypobaric hypoxia (atmospheric pressure 380 mmHg, 10% O_2_). Gr4 (HM) rats received MCT 40 mg/kg and were subjected to hypobaric hypoxia starting on day one. The hypoxia chamber was opened twice per week for 15 min to weigh the rats, replenish food and water, and provide clean bedding similar to the other rats in room air. We did not notice any obvious cyanosis in these rats; however, increased hemoglobin levels were reported. At the end of four weeks, these rats were studied. All chemicals were purchased from Sigma Aldrich, St Louis, MO, USA.

### 2.2. Measurement of Right-Ventricular Systolic Pressure (RVSP)

As previously described [36], rats were anesthetized with an intraperitoneal injection of xylazine (6 mg/kg) and ketamine (60 mg/kg). Through an incision in the neck, the trachea was exposed and cannulated with PE 240 tubing, and the rat ventilated in room air (~70–80 breaths/min). The chest was opened, PE 50 tubing with a small needle was inserted into the right ventricle, and the pressure was recorded on a Grass polygraph (model 7E). At the end of the pressure measurements, the lungs were perfused with autoclaved normal saline to remove blood. The left lung and the heart were placed in 10% buffered formaldehyde. The right lung was quickly frozen in liquid nitrogen and stored at −80 °C for transcriptomic analysis at a later date.

### 2.3. Assessment of Right-Ventricular Hypertrophy (RVH)

One week later, the heart was removed from formaldehyde and the atria were trimmed. The free wall of the right ventricle (RV) was separated from the left ventricle (LV) and the septum (S) and weighed. The RVH was calculated for all groups as follows:

(1) $$RVH^{\left(PH\right)}\equiv \bar{{\left(\frac{RV}{LV+S}\right)}_{weight}^{\left(PH\right)}}/\bar{{\left(\frac{RV}{LV+S}\right)}_{weight}^{\left(CO\right)}}\;,\;\forall (PH)=(HO),(CM),(HM)$$

Right-ventricular hypertrophy occurs when the average RV/(LV + S)(PH)) is significantly (*p* < 0.05) larger than RV/(LV + S)(CO) (i.e., RVH significantly larger than one).

### 2.4. Histology

Formalin-preserved lung tissue was processed for paraffin block. Then, 5–6-μm sections were cut and stained with hematoxylin and eosin (H&E) for evaluation of the pulmonary vasculature.

### 2.5. Microarray

Lungs were quickly removed, frozen in liquid nitrogen, and stored at −80 °C. Our optimized protocol [37,38] and the “multiple yellow” strategy [39] were used to profile each “condition” in four biological replicas. Briefly. total RNA was extracted with Qiagen RNeasy minikit, concentration was determined with a NanoDrop ND-2000 Spectrophotometer, and purity determined with an Agilent RNA 6000 Nano kit in an Agilent 2100 Bioanalyzer. Total RNA was then reverse-transcribed in the presence of Cy3/Cy5 dUTP and the incorporation of fluorescent tags was determined again with the nanodrop. Differently labeled samples of biological replicates were hybridized overnight with Agilent 60 mer 4 × 44k whole genome rat V2 arrays (#G2519F). The arrays were scanned with an Agilent G2539A dual-laser scanner, and primary analysis was performed with (Agilent) Feature Extraction 11.1 software. All spots affected by local corruption or with foreground fluorescence less than twice the background were disregarded, and data were normalized and filtered following our standard procedure [40].

### 2.6. Transcriptomic Analysis

A gene was considered as significantly regulated if the absolute fold-change exceeded the cut-off calculated for that gene [41]. The cut-off (CUT, Equation (1)) accounts for the observed expression variability of that gene in the compared conditions and the existence of several spots probing it redundantly in the microarray. The non-uniform expression variability among the genes results from both the biological variability within biological replicas and the inherent technical noise. This strategy aims to substantially reduce both the positive and the negative false hits when using an arbitrary cut-off (e.g., 1.5×) for the fold-change. CUT (Equation (1)) adds correction for the groups of microarray spots redundantly probing the same gene.

(2) $$\begin{array}{l} CUT_{i}^{\left(PH\right)}=1+\frac{1}{2}\underset{\mathrm{chi}-\mathrm{square}\;\mathrm{correction}}{\underbrace{\left(\sqrt{\frac{r_{i}}{\chi^{2}\left(r_{i};1-\varepsilon /2\right)}}+\sqrt{\frac{r_{i}}{\chi^{2}\left(r_{i};\varepsilon /2\right)}}\right)}}\left(\underset{\mathrm{pooled}\;\mathrm{CV}}{\underbrace{\sqrt{\frac{1}{R_{i}}\sum_{k=1}^{R_{i}}\left({\left(\frac{s_{ik}^{\left(PH\right)}}{\mu_{ik}^{\left(PH\right)}}\right)}^{2}+{\left(\frac{s_{ik}^{\left(CO\right)}}{\mu_{ik}^{\left(CO\right)}}\right)}^{2}\right)}}}\right)\;,\;where:\\ \left(PH)=(HO),(CM),(HM\right)\\ r_{i}=4R_{i}-1{\;=\;\mathrm{number}\;\mathrm{of}\;\mathrm{degrees}\;\mathrm{of}\;\mathrm{freedom},\;R}_{i}\;=\;\mathrm{number}\;\mathrm{of}\;\mathrm{spots}\;\mathrm{probing}\;\mathrm{the}\;\mathrm{same}\;\mathrm{gene}\;i\\ \chi^{2}\;=\;\mathrm{chi}-\mathrm{square}\;\mathrm{score}\;\mathrm{for}\;r_{i}\;\mathrm{degrees}\;\mathrm{of}\;\mathrm{freedom}\;\mathrm{and}\;\mathrm{probability}\;\varepsilon \;(=0.05)\;\mathrm{in}\;\mathrm{this}\;\mathrm{report}\\ s_{ik}^{\left(condition\right)}=\;\mathrm{standard}\;\mathrm{deviation}\;\mathrm{of}\;\mathrm{gene}\;i\;\mathrm{probed}\;\mathrm{by}\;\mathrm{spot}\;k\;\mathrm{in}\;\mathrm{the}\;\mathrm{specified}\;\mathrm{condition}\\ \mu_{ik}^{\left(condition\right)}\;=\;\mathrm{average}\;\mathrm{expression}\;\mathrm{of}\;\mathrm{gene}\;i\;\mathrm{probed}\;\mathrm{by}\;\mathrm{spot}\;k\;\mathrm{in}\;\mathrm{the}\;\mathrm{specified}\;\mathrm{condition} \end{array}$$

Profiling four biological replicates allows independent assessment of (i) average expression level, (ii) expression variability, and (iii) expression coordination of each gene with each individual gene. Combination of these measures was used to determine the remodeling of the topology and interplay of the functional genomic fabrics. The genomic fabric was defined by us as the most inter-coordinated and stably expressed gene network responsible for selected biological processes [42].

### 2.7. Pathway Analysis

The genes pertaining to important functional pathways were selected from the maps developed by Kyoto Encyclopedia of Genes and Genomes (KEGG) [43]. Particular attention was given to the genes involved in oxidative phosphorylation (map 00190, ATPases, cytochrome c oxidases, NADH dehydrogenases), chemokine signaling pathway (map 04062), vascular smooth muscle contraction (map 04270), mitochondrion ribosomal proteins, and cell cycle (map 04110).

The corrected weighted pathway regulation (WPR) [44] was used to quantify the transcriptomic effects of the three PH models on the selected functional pathways.

(3) $$\begin{array}{l} WPR_{\Gamma }^{\left(PH\right)}={\langle \mu_{i}^{\left(CO\right)}\underset{=0\;\mathrm{if}\;\left|x_{i}^{\left(PH\right)}\right|<CUT_{i}^{\left(PH\right)}}{\underbrace{\left(\left|x_{i}^{\left(PH\right)}\right|-CUT_{i}^{\left(PH\right)}\right)}}\times \underset{\mathrm{confidence}\;\mathrm{of}\;\mathrm{significant}\;\mathrm{regulation}}{\underbrace{\left(1-p_{i}^{\left(PH\right)}\right)}}\rangle }_{\mathrm{all}\;i\in \Gamma }\;,\;where:\;\mathrm{PH}\;=\;\mathrm{CM},\;\mathrm{HO}\;\mathrm{or}\;\mathrm{HM}\\ \langle A\rangle \;=\;\mathrm{median}\;\mathrm{of}\;A\;\mathrm{for}\;\mathrm{all}\;\mathrm{genes}\;\mathrm{within}\;\mathrm{the}\;\mathrm{functional}\;\mathrm{pathway}\;\Gamma \\ x_{i}^{\left(PH\right)}\;=\;\mathrm{fold}-\mathrm{change}\;(\mathrm{negative}\;\mathrm{for}\;\mathrm{down}-\mathrm{regulation}\;\mathrm{in}\;\mathrm{the}\;\mathrm{PH}\;\mathrm{model}\;\mathrm{with}\;\mathrm{respect}\;\mathrm{to}\;\mathrm{CTR}\\ p_{i}^{\left(PH\right)}\;=\;p-\mathrm{value}\;\mathrm{of}\;\mathrm{the}\;\mathrm{expression}\;\mathrm{regulation}\;\mathrm{of}\;\mathrm{gene}\;i\;\mathrm{in}\;\mathrm{the}\;\mathrm{PH}\;\mathrm{model} \end{array}$$

WPR is more informative than the traditional percentage of regulated genes. WPR weights the contribution of each gene proportional to its normal (here, in the CO group) average expression, absolute fold-change (until the cut-off accounting for the combined contributions of the technical noise and biological variability), and confidence of the expression regulation.

Like in previous experiments with kidneys [45] and hearts of mice subjected to chronic hypoxia [39,46,47], we computed the pair-wise Pearson correlation coefficient *ρij* between the expression levels of all pairs of expressed genes i and j in biological replicas of each condition. As speculated in previous papers [48,49], strong expression correlation of two genes may represent a kind of “transcriptomic stoichiometry” aiming to optimize the pathway involving the encoded proteins. Expressions of (*p* < 0.05) statistically significantly synergistically expressed genes (*ρij* > 0.950) oscillate in phase (both genes increase and decrease their expression simultaneously), while expressions of antagonistically expressed genes (*ρij* < −0.950) manifest opposite tendencies (when one increases, the other decreases). Genes with |*ρij*| < 0.025 are considered independently expressed. Different from the traditional cluster analysis that correlates the genes according to their similar behavior across various conditions or time points, we compute the correlation only between the expression levels in biological replicas of the same condition to determine “stoichiometric gene networks” [49].

The influence of a given gene i toward a particular pathway Γ was evaluated by its average coordination power (CP) against the expressions of the pathway Γ genes.

(4) $$CP_{i;\Gamma }^{\left(condition\right)}={\langle \rho_{ij}^{\left(condition\right)}\rangle }_{j\in \Gamma }\times 100/\%.$$

CP takes values from −100% (all Γ-genes perfectly negatively correlated with gene i) to 100% (all Γ-genes perfectly positively correlated with gene i). A neutral gene toward that pathway has the CP close to zero, indicating that either almost all correlations are null or the positives are balanced by the negatives.

Here, we introduce a new measure, termed gene pair prominence (GPP), to quantify the relevance of gene interplays in each condition. The two genes can be from the same or from two different functional pathways. GPP takes values from −100% for the most prominent, negatively correlated genes to 100% for the most prominent, positively correlated genes in that condition. In order to simplify the landscape, GPP was considered zero for gene pairs whose absolute Pearson product-moment correlation coefficient between their expression levels in the four biological replicas of that condition was not significant (|*ρ_ij_*| < 0.950).

(5) $$\begin{array}{l} GPP_{ij}^{\left(condition\right)}=\{\begin{array}{lll} \frac{\mu_{i}^{\left(condition\right)}\rho_{ij}^{\left(condition\right)}\mu_{j}^{\left(condition\right)}}{\max{\left|\mu_{i}^{\left(condition\right)}\rho_{ij}^{\left(condition\right)}\mu_{j}^{\left(condition\right)}\right|}_{i\in \Gamma_{1}j\in \Gamma_{2}}}\times 100\% & if & \left|\rho_{ij}^{\left(condition\right)}\right|>0.950\\ 0 & if & \left|\rho_{ij}^{\left(condition\right)}\right|<0.950 \end{array}\;,\;where:\\ \rho_{ij}^{\left(condition\right)}\;\mathrm{is}\;\mathrm{the}\;\mathrm{pair}-\mathrm{wise}\;\mathrm{Pearson}\;\mathrm{correlation}\;\mathrm{coefficient}\;\mathrm{of}\;\mathrm{the}\;\mathrm{expression}\;\mathrm{levels}\;\mathrm{of}\;\mathrm{genes}\;i\in \Gamma_{1},\;j\in \Gamma_{2}\\ condition\;=\;\mathrm{CO},\;\mathrm{HO},\;\mathrm{CM},\;\mathrm{HM};\;\Gamma_{1},\Gamma_{2}\;=\;\mathrm{functional}\;\mathrm{pathways};\;|X|\;=\;\mathrm{absolute}\;\mathrm{value}\;\mathrm{of}\;X \end{array}$$

## 3. Results

### 3.1. Decreased Weight Gain

**Figure 1:**
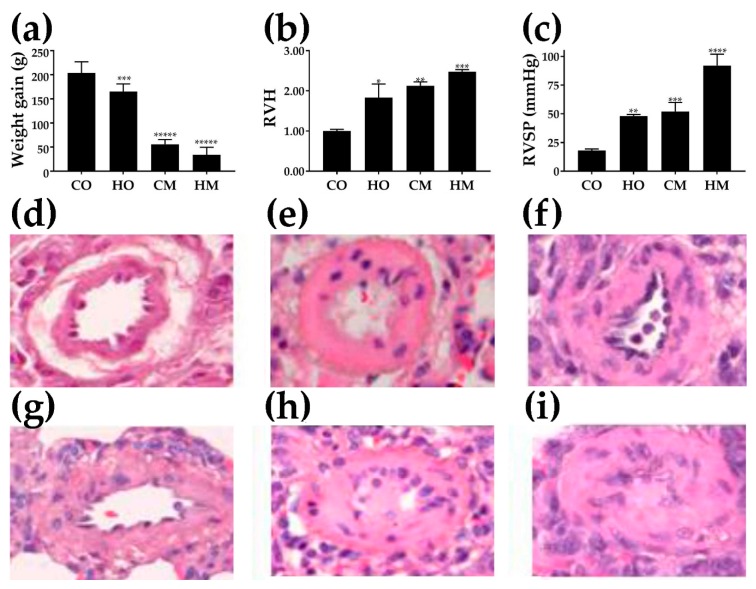
Effects of the pulmonary hypertension on (**a**) weight gain (*n* = 8), (**b**) right-ventricle hypertrophy (RVH, dimensionless measure) (*n* = 8), (**c**) right-ventricle systolic pressure (RVSP) (*n* = 8), and (**d**–**i**) histology of the pulmonary arteries. Hematoxylin and eosin (H&E)-stained sections from lungs of rats from the groups: control (CO) (**d**), hypoxia (HO) (**e**), monocrotaline (CM) (**f**), and monocrotaline and hypoxia (HM) (**g**–**i**). Note in (**h**) and (**i**) that the pulmonary arteries exhibit luminal obstruction. Statistical significance (*t*-test): * (*p* < 0.05), ** (*p* < 0.01), *** (*p* < 0.005), **** (*p* < 0.001), ***** (*p* < 0.0001).

The four-week weight gain in the control group was (204 ± 23) g. All PH groups had significantly lower weight gains as presented in Figure 1a; the lowest was observed in MCT-treated rats exposed to hypoxia, i.e., group HM (WG = 33.6 ± 16.0) g.

### 3.2. Right-Ventricle Hypertrophy

Figure 1b presents the significant increase in ratio between the right-ventricle weight and the sum of the left-ventricle and septum weights in the three PH models as normalized to the RV/(LV + S) in the control group. Note that right-ventricle hypertrophy was statistically significant in all three experimental models.

### 3.3. Increased Right-Ventricle Systolic Pressure

As depicted in Figure 1c, RVSP was significantly increased in all PH groups (HO, CM, HM) compared to the controls (CO). While the difference between HO and CM groups was not statistically significant (*p* = 0.364), the differences between the HM and HO groups (*p* = 0.000127) and between HM and CM groups (*p* = 0.00078) were highly significant.

### 3.4. Histological Alterations

Figure 1d–i present H&E-stained pulmonary arteries (external diameter, 42–101 μm) from the controls (D) and the experimental groups: HO (e), CM (f), and HM (g, h, i). Pulmonary arteries from CM and HO show substantial medial wall thickening compared with the control. The arteries from the HM group show further medial wall thickening, and as seen in (h) and (i), exhibit neointima formation and occlusion of the lumen.

### 3.5. Overview of the Transcriptomic Alterations

**Figure 2:**
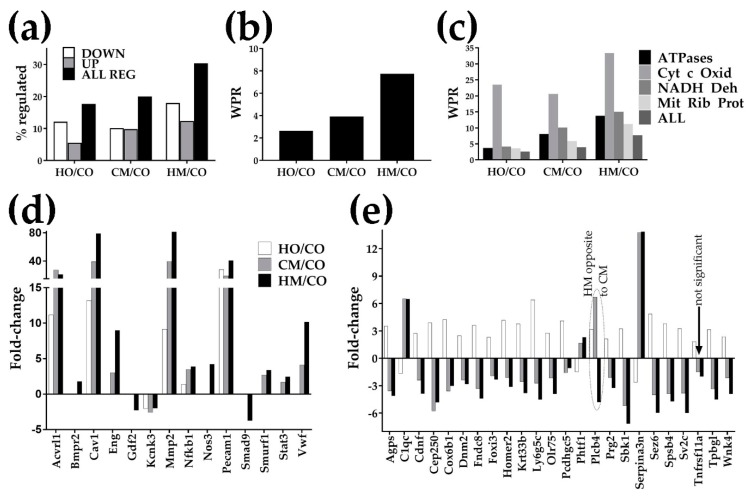
Transcriptomic alterations: (**a**) number of regulated genes; (**b**) weighted pathway regulation of all genes; (**c**) weighted pathway regulation for some important groups of genes. Pathways: Cyt c Oxid = cytochrome c oxidases, NADH Deh = NADH dehydrogenases, Mit Rib Prot = mitochondrial ribosomal proteins. Note that Cyt c Oxid was by far the most altered group of respiratory genes. HO/CO, CM/CO, HM/CO = expression fold-change (negative for downregulation) in the indicated comparison; (**d**) statistically significant fold-changes of genes whose altered structure and/or expression is often associated with PH. Genes: *Acvrl1* (activin A receptor type II-like 1), *Bmpr2* (bone morphogenetic protein receptor, type II (serine/threonine kinase)), *Cav1* (caveolin 1, caveolae protein), *Eng* (endoglin), *Gdf2* (growth differentiation factor 2), *Kcnk3* (potassium channel, subfamily K, member 3), *Mmp2* (matrix metallopeptidase 2), *Nfkb1* (nuclear factor NF-kappa-B p105 subunit), *Nos3* (nitric oxide synthase 3, endothelial cell), *Pecam1* (platelet/endothelial cell adhesion molecule 1), SMAD9 (SMAD family member 9), *Smurf1* (SMAD-specific E3 ubiquitin protein ligase 1), *Stat3* (signal transducer and activator of transcription 3 (acute-phase response factor)), *Vwf* (von Willebrand factor); (**e**) significant opposite regulations. Genes: *Agps* (alkylglycerone phosphate synthase), *C1qc* (complement component 1, q subcomponent, C chain), *Cdnf* (cerebral dopamine neurotrophic factor), *Cep250* (centrosomal protein 250), *Cox6b1* (cytochrome c oxidase subunit 6B1), *Dnm2* (dynamin 2), *Fndc8* (fibronectin type III domain-containing 8), *Foxi3* (forkhead box I3), *Homer2* (homer homolog 2), *Krt33b* (keratin 33B), *Ly6g5c* (lymphocyte antigen 6 complex, locus G5C), *Olr75* (olfactory receptor 75), *Pcdhgc5* (protocadherin γ subfamily C, 5), *Phtf1* (putative homeodomain transcription factor 1), *Plcb4* (phospholipase C, β 4), *Prg2* (proteoglycan 2, bone marrow), *Sbk1* (SH3-binding domain kinase 1), *Serpina3n* (serine), *Sez6* (seizure-related 6 homolog), *Spsb4* (splA/ryanodine receptor domain and SOCS box-containing 4), *Sv2c* (synaptic vesicle glycoprotein 2c), *Tnfrsf11a* (tumor necrosis factor receptor superfamily, member 1a), *Tpbgl* (trophoblast glycoprotein-like), *Wnk4* (WNK lysine-deficient protein kinase 4).

Raw and processed gene expression results were deposited and are publicly available at [50]. Figure 2a,b indicate that all three experimental PH conditions regulated large numbers of genes and produced large weighted pathway regulation (WPR) scores. The overall transcriptomic alterations (% of ALL REG and WPR) were consistent with the alterations in weight gain, RVH, and RVSP data. Thus, MCT alone had a slightly larger effect than hypoxia alone; when MCT-treated rats were exposed to hypoxia, the alterations were substantially larger. Figure 2c presents the WPR scores of some interesting groups of genes involved in respiration. Figure 2d presents the significant fold-changes of genes whose regulation is often associated with PH. Remarkably, in all three rat models of PH, *Cav1* was overexpressed, by 13.18× in HO, 39.14× in CM, and 79.06× in HM, with a substantial increase in the HM group. We found also that upregulation of *Cav1* was accompanied by the upregulation of *Nos3* (by 4.21× in HM) and upregulation of *Mmp2* (by 9.14× in HO, 33.59× in CM, and 81.47× in HM group). The vast majority of the regulated genes by separate exposure to either hypoxia or MCT were altered in the same direction. However, as presented in Figure 2e, few of them were oppositely regulated. Interestingly, when hypoxia and MCT were combined (as in HM), the regulation mostly followed the effects of the MCT exposure alone but with a larger fold-change (except for *Plcb4* whose regulation in the HM group was opposite to regulations in both HO and CM groups).

### 3.6. Regulation of the Immune-Inflammatory Response

We found that PH regulated the expression of numerous genes involved in the (KEGG-determined map 04062) chemokine signaling pathway (Table 1). Table S1 in the Supplementary presents other immune-inflammatory response genes (chemokines, cytokines, cytokine receptors, interferons, interleukins, and tumor necrosis factors) that were significantly regulated in at least one of the three rat PH models. Importantly, compared to the traditional standard of uniform 1.5× absolute fold-change, our procedure to attach fold-change cut-offs to every single gene in each comparison identified additional regulated genes and eliminated false hits.

### 3.7. Regulation of the Vascular Smooth Muscle Contraction Pathway

**Figure 3:**
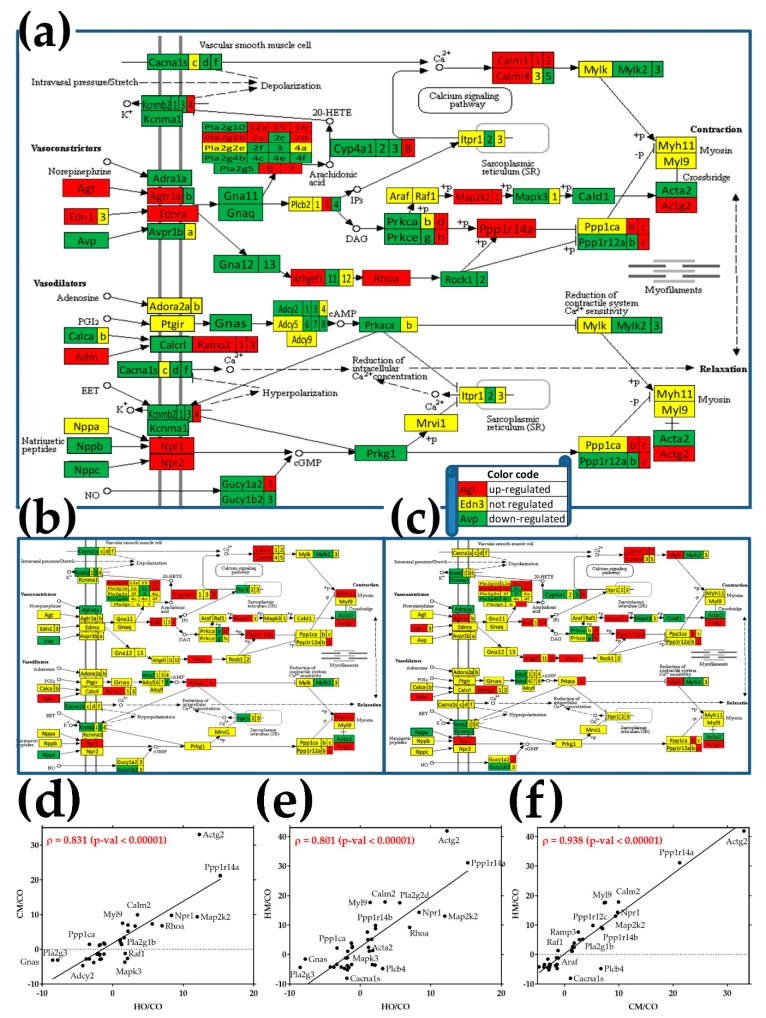
Regulation of the vascular smooth muscle contraction pathway. (**a**) Rats treated with monocrotaline (MCT) and exposed to hypoxia (HM); (**b**) rats exposed to only hypoxia (HO); (**c**) rats treated with monocrotaline only (CM). Regulated genes: *Acta2* (smooth muscle α-actin), *Actg2* (actin, γ 2, smooth muscle, enteric), *Adcy2* (adenylate cyclase 2), *Adora2a* (adenosine A2a receptor), *Agtr1a* (angiotensin II receptor, type 1a), *Ang* (angiogenin), *Araf* (v-raf murine sarcoma 3611 viral oncogene homolog), *Avp* (arginine vasopressin), *Cacna1d/s* (calcium channel, voltage-dependent, L type, α 1D/S subunit), *Cald1* (caldesmon 1), *Calm2* (calmodulin 2), *Cyp4a1/8* (cytochrome P450, family 4, subfamily a, polypeptide 1/8), *Gnas* (GNAS complex locus), *Gucy1b2* (guanylate cyclase 1, soluble, β 2), *Itpr1* (inositol 1,4,5-trisphosphate receptor, type 1), *Kcnmb2* (potassium large conductance calcium-activated channel, subfamily M, β member 2), *Map2k2* (mitogen-activated protein kinase kinase 2), *Mapk3* (mitogen-activated protein kinase 3), *Myl9* (myosin, light chain 9, regulatory), *Mylk2* (myosin light chain kinase 2), *Npr1/2* (natriuretic peptide receptor guanylate cyclase A/B), *Pla2g1b* (phospholipase A2, group IB, pancreas), phospholipases A2 (*Pla2g2d*, *Pla2g3*, *Pla2g4b*, *Pla2g6*), *Plcb4* (phospholipase C, β 4), *Ppp1ca* (protein phosphatase 1, catalytic subunit, α isozyme), *Ppp1r12c/14a/14b* (protein phosphatase 1, regulatory subunit 12C/14A/14B), *Prkca/e* (protein kinase C, α/epsilon), *Ptgir* (prostaglandin I2 (prostacyclin) receptor (IP)), *Raf1* (v-raf-leukemia viral oncogene 1), *Ramp1/3* (receptor (G protein-coupled) activity modifying protein 1/3), *Rhoa* (Ras homolog family member A); (**d**) correlation of fold-changes of VSMS genes in CM and HO with respect to CO; (**e**) correlation of fold-changes of vascular smooth muscle cell (VSMC) genes in HM and HO with respect to CO; (**f**) correlation of fold-changes of VSMC genes in HM and CM with respect to CO. Note the highly significant correlation of VSMC genes alterations in the three pulmonary hypertension (PH) models. Only genes with significant regulation in at least one PH model were included. Gene symbols were attached where the space allowed good readability.

Figure 3 presents the regulation of the 122 quantified genes involved in the (KEGG-determined map 04270) vascular smooth muscle contraction pathway in the three PH models. Note the substantially increased number of regulated genes when the MCT-treated rats were exposed to hypoxia (30% up + 49% down, Figure 3a) compared to when they were exposed to only hypoxia (16% up + 14% down, Figure 3b) or treated with MCT (26% up + 16% down, Figure 3c). The percentages of all regulated genes in this pathway (30% in HO, 42% in CM, and 79% in HM) were notably larger than the percentages of all regulated genes in the entire transcriptome (18% in HO, 20% in CM, and 30% in HM) as illustrated in Figure 2a. Panels 3d, 3e, and 3f present the statistically significant (*p* < 0.00001) correlations of the fold-changes of the VSMC genes in the three PH models. The analysis revealed a larger Pearson correlation coefficient between the regulation of VSMC genes in CM and HM (ρ = 0.938) than between CM and HO (ρ = 0.831), and HM and HO (ρ = 0.801).

### 3.8. Regulation of the Cell-Cycle Pathway

**Figure 4:**
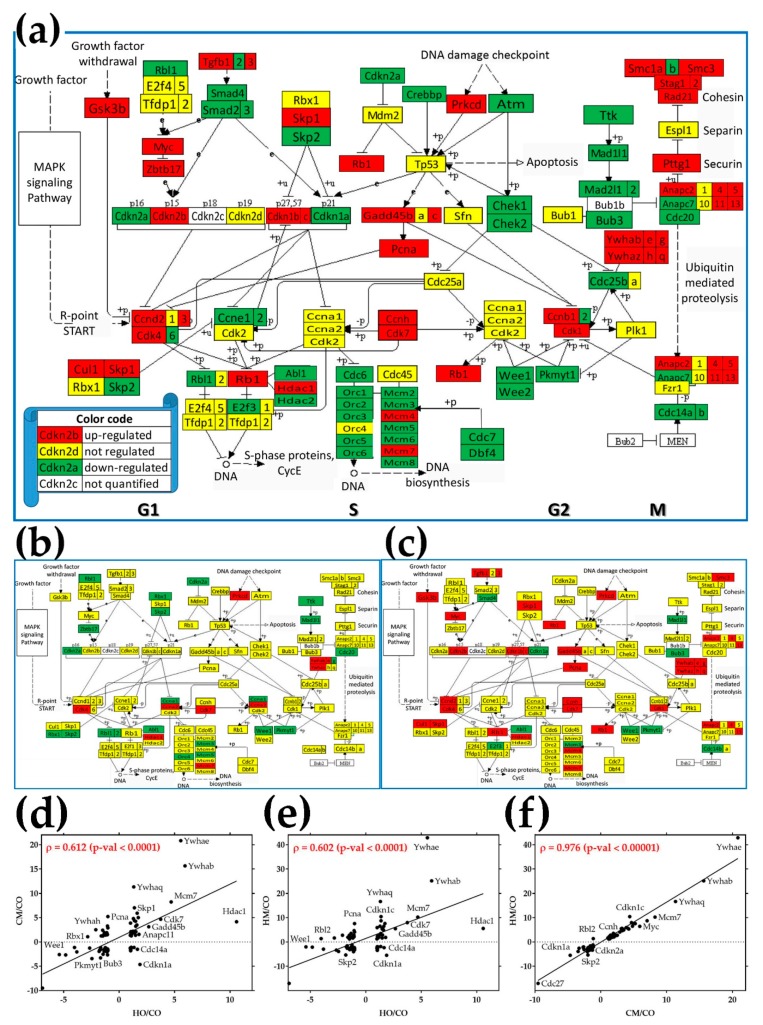
Regulation of the cell-cycle (CC) pathway. (**a**) Rats treated with MCT and exposed to hypoxia (HM); (**b**) rats exposed to only hypoxia (HO); (**c**) rats treated with monocrotaline only (CM). Regulated genes: *Abl1* (C-abl oncogene 1, non-receptor tyrosine kinase), subunits of the anaphase promoting complex (*Anapc2, Anapc4, Anapc5, Anapc7, Anapc11, Anapc13*), *Atm* (ataxia telangiectasia mutated homolog), *Bub3* (budding uninhibited by benzimidazoles 3 homolog), cyclins (*Ccnb1, Ccnb2, Ccnd2, Ccnd3, Ccne1, Ccne2, Ccnh*), cell division cycles (*Cdc14a, Cdc14b, Cdc20*, *Cdc25a, Cdc25b, Cdc6*), cyclin-dependent kinases (*Cdk1, Cdk4, Cdk6, Cdk7*), cyclin-dependent kinase inhibitors (*Cdkn1a, Cdkn1b, Cdkn1c, Cdkn2a, Cdkn2b*), checkpoint kinases (*Chek1, Chek2*), *Cul1* (culin 1), *Dbf4* (DBF4 homolog), *Gsk3b* (glycogen synthase kinase 3 β), *E2f3* (E2F transcription factor 3), growth arrest and DNA-damage-inducible (*Gadd45b, Gadd45c*), histone deacetylases (*Hdac1, Hdac2*), MAD2 mitotic arrest deficient-likes (*Mad2l1, Mad2l2*), components of the minichromosome maintenance complex (*Mcm2, Mcm3, Mcm4, Mcm5, Mcm6, Mcm7, Mcm8*), *Myc* (myelocytomatosis oncogene), subunits of the origin recognition complex (*Orc1, Orc2, Orc3,, Orc4, Orc5, Orc6*), *Pcna* (proliferating cell nuclear antigen), *Pkmyt1* (protein kinase, membrane associated tyrosine/threonine 1), *Prkcd* (protein kinase C, delta), *Rad21* (RAD21 homolog), retinoblastomas (*Rb1, Rbl1*), *Skp1/2* (S-phase kinase-associated protein 1/2), structural maintenance of chromosomes (*Smc1a, Smc1b, Smc3*), SMAD family members (*Smad2, Smad3, Smad4*), stromal antigens (*Stag1, Stag2*), transforming growth factors β (*Tgfb1, Tgfb2, Tgfb3*), *Wee1* (WEE1 G2 checkpoint kinase), *Wee2* (oocyte meiosis inhibiting kinase), tyrosine 3-monooxygenase/tryptophan 5-monooxygenase activation proteins (*Ywhab, Ywhae, Ywhag, Ywhah, Ywhaq, Ywhaz*), *Zbtb17* (zinc finger and BTB domain-containing 17); (**d**) correlation of fold-changes of CC genes in CM and HO with respect to CO; (**e**) correlation of fold-changes of CC genes in HM and HO with respect to CO; (**f**) correlation of fold-changes of CC genes in HM and CM with respect to CO. Note the highly significant correlation of VSMC genes alterations in the three PH models. Only genes with significant regulation in at least one PH model were included. Gene symbols were attached where the space allowed good readability.

Figure 4 presents the regulation of the 112 quantified genes responsible for the (KEGG-determined map 04110) cell-cycle pathway in the three PH models with respect to control. As expected, many more genes were regulated in the lungs of MCT-treated rats exposed to hypoxia (36% up + 42% down, Figure 4a) than in those of rats exposed to only hypoxia (7% up + 16% down, Figure 4b) or treated with monocrotaline alone (25% up + 11% down, Figure 4c). Of note are the much higher percentages of regulated genes in the cell-cycle pathway (23% in HO, 36% in CM and 78% in HM) than the percentages of regulated genes in the entire transcriptome (18% in HO, 20% in CM and 30% in HM) as illustrated in Figure 2a.

Genes were regulated in both DNA replication phase (S) and mitosis phase (M), as well as during the temporal separation gaps (denoted by G1 and G2 in Figure 4a). Interestingly, all components of the minichromosome maintenance complex (*Mcm*) were regulated in MCT-treated rats exposed to hypoxia, with downregulation of *Mcm3* and upregulation of *Mcm7* consistent in the other two PH groups. In contrast, while no subunit of the origin recognition complex (*Orc*) was regulated by MCT administration and only *Orc4* was downregulated by hypoxia, when MCT-treated animals were exposed to hypoxia, all but *Orc4 Orc* subunits were regulated. Panels 4d, 4e, and 4f present the statistically significant correlations of the fold-changes of the CC genes in the three PH models. The analysis revealed a larger Pearson correlation coefficient between the regulation of CC genes in CM and HM (ρ = 0.976) than between CM and HO (ρ = 0.602) or HM and HO (ρ = 0.602).

### 3.9. Alteration of Cellular Respiration

Particular attention was given to the regulation of the major groups of genes involved in the active ionic transport across the plasma membrane (ATPases) and oxidative phosphorylation: cytochrome c oxidases and NADH dehydrogenases (Table 2). As illustrated In Figure 2c, the weighted pathway regulation (WPR) score revealed that these groups of genes were altered much more than the entire transcriptome. Remarkably, *Atp1b2*, one of the major players responsible for establishing and maintaining the transmembrane electrochemical gradients of Na^+^ and K^+^, was the most downregulated in all three PH models (−146× in HO, −257× in CM, and −111× in HM).

Alteration of the genes encoding mitochondrial ribosomal proteins is also presented for comparison in the Supplementary Table S2.

### 3.10. Remodeling of the Relationship between the Genes Involved in the Vascular Smooth Muscle Contraction and Oxidative Phosphorylation

**Figure 5:**
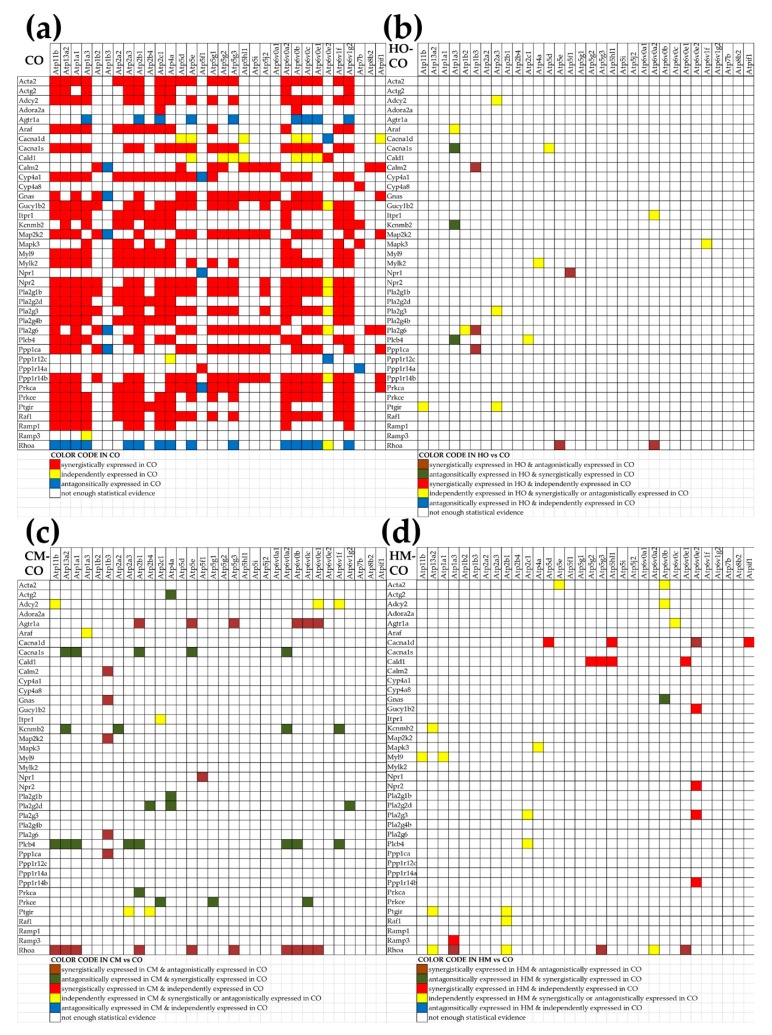
Synergistically (red squares), antagonistically (blue squares), and independently (yellow squares) expressed pairs of VSMC genes and ATPases in CO (**a**) whose expression correlation was significantly altered by hypoxia (**b**), monocrotaline (**c**), or the combined action of hypoxia and monocrotaline (**d**). A blank square in (**a**) indicates that the correlation is not (*p* < 0.05) statistically significant.

We studied how much the interaction of the vascular smooth muscle contraction genes (VSMC, rno04270) (presented in Figure 3) with genes involved in oxidative phosphorylation (OP), particularly with ATPases, is remodeled by pulmonary hypertension. For this, we computed the Pearson correlation coefficients for the 1248 pairs that can be formed with the 32 ATPases and 39 VSMC genes adequately quantified in each of the four experimental conditions. Interestingly, we found that each PH condition significantly reduced the net positive correlation (i.e., number of positively (p) − number of negatively (n) correlated gene-pairs): from 450 (= 472p − 22n) in CO to 236 (= 280p − 44n) in HO, 340 (= 455p − 115n) in CM and 208 (= 262p − 54n) in HM. Figure 5 presents the significantly synergistically, antagonistically, and independently expressed pairs of VSMC genes with ATPases in CO, and the pairs whose expression correlation was significantly altered in the three PH models. Interestingly, the negative correlation of *Rhoa* with 15 ATPases (*Atp11b, Atp13a2, Atp1a1, Atp1a3, Atp2a3, Atp2b1, Atp2c1, Atp4a, Atp5e, Atp5g3, Atp6v0a2, Atp6v0b, Atp6v0c, Atp6v0e1, Atp6v1g2*) and no positive correlation in control rats was cancelled in the PH models, with several correlations even reversed. 

We then used the new measure gene pair prominence (GPP, Equation (4)) to rank the gene pairs of the interplay between the two groups of genes. The positive and negative GPPs were plotted separately (as CO+ and CO−, HO+ and HO−, CM+ and CM−, HM+ and HM−) to emphasize the type and importance of their expression correlation. The GPPs of all genes in each condition were expressed as the percentage of the maximal absolute GPP in that condition, regardless of whether it was positive or negative. Except for the CM condition, where the genes of the dominant pair (*Cyp4a8-Atp5e*) were negatively correlated, the positively correlated pairs had much larger GPPs than the negatively correlated ones, meaning that expression of most ATPases oscillates in phase with most VSMC genes.

The analysis pointed out three remarkable H^+^ transporters (*Atp5e, Atp6v0a2, Atp6v0e2*) in strong partnership with three VSMC genes: *Cyp4a8* (cytochrome P450 family 4, subfamily a, polypeptide 8), *Gnas* (GNAS complex locus), and *Mapk3* (mitogen-activated protein kinase 3)*. Mapk3*, involved in a wide range of pathways (listed in [51]), provides the most prominent positive pairs with *Atp6v0a2* in normal atmospheric conditions and with *Atp6v0e2* in hypoxia (obtained by reducing the atmospheric pressure in half).

## 4. Discussion

We examined the transcriptomic alterations in the right lungs of male rats with pulmonary hypertension induced by exposure to hypoxia (HO group), administration of monocrotaline (CM group), or exposure to hypoxia of monocrotaline-treated animals (HM group). There are numerous common features of the three experimental groups, making them valuable models for the human pulmonary hypertension, including lower weight gain but right-ventricle hypertrophy and increased right-ventricle systolic pressure. However, there are also significant differences as revealed by the present and previous studies.

At four weeks after hypobaric hypoxia, there is significant PH and RVH. The hypoxia-induced PH in rats is not associated with any disruption of EC, loss of endothelial Cav1 or Nos3, or enhanced expression of Cav1 in VSMC. Despite the presence of endothelial Cav1 protein, pro-proliferative and anti-apoptotic pathways are activated, indicating Cav1 dysfunction [30,52]. In addition, the lung sections from infants with respiratory distress syndrome show that the presence of increased pulmonary artery pressure unaccompanied by endothelial damage does not result in the loss of endothelial CAV1 or enhanced expression of *CAV1* in VSMC [29].

In the CM model, the loss of endothelial Cav1 is accompanied by the loss of endothelial proteins such as Pecam1 and soluble guanylate cyclase, and the activation of pro-proliferative and anti-apoptotic pathways leading to PH. At two weeks post monocrotaline administration, progressive loss of endothelial Cav1 is associated with PH and RVH in rats [12,40]. As the disease progresses, a further loss of endothelial Cav1 is accompanied by a loss of von Willebrand factor (vWF), indicative of extensive endothelial damage. Importantly, some of the arteries with vWF loss start to exhibit enhanced expression of CAV1 in VSMC, accompanied by increased *Mmp2* expression and activity [51]. At four weeks, the significant loss of endothelial Cav1 is accompanied by enhanced expression of *Cav1* in VSMC. The total *Cav1* expression in the lungs increases by 39.14× compared to controls. 

Exposing monocrotaline-treated rats to hypoxia (HM group) accelerates the disease process, resulting in significantly higher RVSP and RVH, extensive EC damage, and endothelial Cav1 loss, accompanied by enhanced expression of Cav1 in VSMC and neointima lesions [36,53,54]. Furthermore, 61% of the arteries exhibited enhanced expression of *Cav1* in VSMC; *Cav1* expression in the lungs increased to 81% of the controls. However, the neointimal lesions in the HM group exhibited loss of Cav1 and normal *Nos3* expression. In the absence of Cav1, Nos3 gets uncoupled and produces nitrosative and oxidative stress. *Cav1* expression is significantly increased (79.06×). It is worthy of note here that the neointimal cells have low *Cav1* and near normal *Nos3* expression, which is a set-up for oxidative and nitrosative stress. These results strongly support a dual role of Cav1 in the pathogenesis and the progression of PH, similar to what was reported in cancer [55,56].

We found that *Mmp2* was overexpressed by 9.14× in HO, by 39.59× in CM, and by 81.47× in HM. MMP2 is known to degrade extracellular matrix and facilitate cell proliferation and migration. Increased *MMP2* expression activity was reported in human PAH [57,58] and in monocrotaline-induced PH in rats [53].

Many of the upregulated inflammatory genes from Table 1 were also reported by other authors as related to PH. For instance, *Ccl5* (also known as RANTES; 4.56× in CM and 4.40× in HM), an important chemoattractant for monocytes and T cells, was shown to be increased in PAH [59]. Interestingly, deletion of *Ccl5* was shown to attenuate the Sugen-hypoxia model of PH via Cav1-dependent amplification of Bmpr2 signaling [60]. As presented in Figure 2d, we found *Bmpr2* as upregulated by 1.78× in HM, but it stayed practically unchanged in HO and CM.

Increased expression of CXCR4, a receptor for the chemokine stromal cell-derived factor 1 (SDF1), was reported in the lungs of patients with IPAH, HPAH, and PAH associated with congenital heart defect [61]. Increased Cxcr4 was also reported in Sugen + hypoxia and monocrotaline + hypoxia rodent models of PH [62]. Inhibition of Cxcr4 moderately attenuated pulmonary vascular remodeling in the Sugen + hypoxia model [63], while overexpression of Cxcr4 participates in the repair of tissue injury [64]. In the present study (Table S1), *Cxcr4* was found to be upregulated in CM (4.56×) and HM (2.84×). The lower amplification in HM as compared to CM may be explained by the (even not statistically significant) negative effect of hypoxia (−1.21× in HO). Of note is the upregulation of the tumor necrosis factors and their receptors, confirming the upstream regulator role of the TNF [1].

*Ciapin1* (upregulated by 3.16× in CM and by 5.16× in HM, Table S1) promotes anti-apoptosis and cell proliferation via the cyclins D1 (Ccnd1) and E1 (Ccne1), and cyclin-dependent kinases (Cdk) 2 and 4 [65]. Our study confirmed the upregulation for *Ccnd1* (1.93× in HM) and *Cdk4* (1.49× in HO, 4.26× in CM, and 5.31× in HM). Yet, we found *Cdk2* as not regulated and *Ccne1* as downregulated (−2.78× in HO). Interestingly, in human VSMC cell culture, *CIAPIN1* siRNA was shown to inhibit cell proliferation and enhance apoptosis by increasing *Bcl2* and *Bax* [66]. These results in cell culture suggest that upregulation of *Ciapin1* should correlate with downregulation of *Bcl2* and *Bax.* Indeed, we found *Bcl2* as downregulated (−2.47× in HM), but *Bax* was found as upregulated (2.59× in CM and 2.69× in HM). However, the upregulation of *Bax* is not necessarily a contradiction because *Ciapin1* knockdown by siRNA and its upregulation by MCT are triggered by different mechanisms and, hence, not symmetrical phenomena, involving the opposite regulation of the same set of genes. Moreover, as we proved for oligodendrocytes [67] and neurons [68], the heterogeneous cellular environment and external stimuli (missing in the VSMC monoculture but present in the lung) are potent transcriptome modulators.

Among others, we found that *Nfkb1* (nuclear factor kappa B subunit 1), one of the controllers of the cytokine production, was upregulated by 3.50× in CM and by 3× in HM (Figure 1d). *Icam1* (intercellular adhesion molecule 1) was significantly upregulated by 6.79× in HO, 23.65× in CM, and 43.97× in HM, while *Vcam1* (vascular cell adhesion molecule 1) was upregulated by 2.12× in HO, by 6.46× in CM, and by 11.25× in HM. *Lgals3* (galectin3), with a critical role in vascular inflammation and fibrosis and heart failure, was also found as upregulated by 10.89× in CM and 17.83× in HM. Importantly, increased expression of *Lgals3* and *Icam1* was shown to be present in patients with IPAH and connective tissue disease [69].

*Anxa 1* (annexin A1, increased by 9.64× in HO, 56.48× in CM, and 106.00× in HM) plays an essential role in cell invasion and migration [70]. It is also an anti-inflammatory protein that controls pro-inflammatory mediator release. It promotes leukocyte detachment from EC and serves as a negative regulator of the transmigratory processes [71,72], and it provides protection from neointima formation in atherosclerosis [73]. Eng (endoglin, 3.03× in CM and 9.00× in HM), a transmembrane receptor for TGFβ signaling, plays a key role in the balance of Alk1 and Alk5 signaling that regulates EC proliferation. Increased expression of *Alk1/Eng* was reported in EC in IPAH. Furthermore, endoglin deficiency protects mice from hypoxic PH [74]. *Nfat5* (nuclear factor activated T cells 5; 3.59× in HM), is implicated in the regulation of genes associated with migration and proliferation [75,76]. Our genomic analysis also revealed increased expression of *Edn1* (endothelin 1, 1.88× in HO, 1.83× in CM, and 3.11× in HM), *Myc* (myelocytomatosis oncogene, 5.92× in CM and 6.42× in HM), *Pdgfa* (platelet-derived growth factor α, 2.77× in HO, 4.63× in CM, and 6.39× in HM). 

The complementary effects of the administration of monocrotaline and exposure to hypoxia are perfectly illustrated by the regulation of the six quantified cyclin-dependent kinases, the key regulatory enzymes of the cell cycle. Thus, *Cdkn2a* was downregulated in HO and HM groups but not affected in CM, while *Cdkn1a* was downregulated and *Cdkn1b, Cdkn1c*, and *Cdkn2b* were upregulated in CM and HM groups but not in the HO group. Cyclin-dependent kinases are among the targeted genes for the treatment of pulmonary arterial hypertension [77].

As illustrated in panels (d), (e), and (f) in both Figure 3 (VSMC pathway) and Figure 4 (CC pathway), we found significant correlations between the gene expression regulations in the three models. However, the correlation between the regulations in HM and CM models was much stronger than between CM and HO or between HM and HO. We found 23 genes (Figure 2e) whose regulation in HO was opposite to regulation in both CM and HM, and only one gene (*Plcb4*) whose regulation in HM was opposite to regulation in both CM and HM. Since the HM rats were exposed to both hypoxia and MCT treatment, each upregulating *Plcb4*, the downregulation of this phospholipase C in HM is surprising and deserves further study.

Mitochondria are the major sources of reactive oxygen species (ROS) and highly susceptible to oxidative stress. Increased ROS production and mitochondrial dysfunction occur in a number of diseases including cardiovascular diseases. A significant increase (3.46×) in the expression of *Parp1* (poly (ADP-ribose) polymerase-1) was found in the lungs of rats exposed to both MCT and hypoxia (HM group). Most of the activity of Parp1 is localized in the nucleus. Recent studies showed DNA damage and associated increased expression of *Pparp1* to be important aspects of human PAH. Parp1 is implicated in DNA repair, allowing cell proliferation during stress. Parp1 plays an important role in cellular functions during health and disease [78]. Although, in humans, PARP1 was reported to upregulate *HIF1A, IL6*, and *NFAT5* [79,80], we found only *Nfat5* as upregulated (see above). *Hif1α* was downregulated by −1.72× in HO, by −2.04× in CM, and by −1.59× in HM, and *Il*6 was downregulated by −3.18× in HM.

Upregulation of other important genes includes *Slc2a1* (solute carrier family 2 (facilitated glucose transporter), member 1; 2.94× in HM), *Sod2* (mitochondrial superoxide dismutase; 2.23× in HM), *Hmox1* (hemoxygenase 1; 4.53× in CM and 9.11x in HM), *Gpx1* (glutathione peroxidase 1; 20.48× in HO, 84.91× in CM, and 116.90× in HM), *Txn1* (thioredoxin; 7.09× in HO, 54.27× in CM, and 93.90× in HM), *Nfe2l2* (nuclear factor (erythroid-derived-2)-like-2; 3.13× in CM and 5.97× in HM), *Mfn2* (mitofusin 2; 2.69× in HM), and Nq01 (NAD (P) dehydrogenase quinone 1; 3.50× in CM and 3.97× in HM). *Idh2* (isocitrate dehydrogenase; 4.33× in HO, 16.08× in CM, and 28.92× in HM) is an NADPH-generating enzyme that plays an important role in regulating mitochondrial redox balance and diminishing stress-induced injury [81]. Low Mfn leads to attenuated angiogenic response to VEGF and eNOS [82].

Recent studies in PAH revealed a metabolic shift toward aerobic glycolysis (“Warburg effect”) similar to what was observed in cancer, utilized by proliferating cells for survival. Cav1 was shown to stabilize mitochondria, prevent aerobic glycolysis [35], and negatively regulate NADPH-derived reactive oxygen species [82]. Furthermore, Cav1 modulates *Nrf2* expression [83]. It is an important observation that neointimal cells have low Cav1 and normal eNOS expression, resulting in oxidative and nitrosative stress [30]. Compared with the HO group, in the CM and HM groups, there was significantly increased expression of several antioxidants, such as *Nfe2l2*, *Prdx2* (peroxiredoxin 2; 2.66× in HO, 33.94× in CM, and 46.01× in HM), *Gpx1*, *Gpx2* (4.92× in HO, 23.70× in CM, and 24.47× in HM), *Txn1*, *Sod2*, *Hmox1*, and *Nqo1*. These alterations provide an anti-oxidative and glycolytic milieu for the survival of the proliferating cells. 

The analysis of the expression coordination between respiratory and VSMC genes (Figure 5) revealed several interesting interactions. For instance, in CO, *Rhoa* has no positive partners but has 15 negative partners and one independent partner (i.e., 0/15/1) among the 32 ATPases, with its coordination power being CP = −74.47%. The situation is reversed in all the three PH models: nine positives, no negatives, no independents (9/0/0) with a CP of +61.07% in HO, 23/0/1, a CP of +66.37 in CM, 6/0/3, and a CP of +63.93 in HM. Importantly, 10 of the positively correlated ATPase partners of *Rhoa* in CM were negative partners in CO. This switch from negative to positive correlation of *Rhoa* with the ATPases justifies the reported “role of the Nox4-derived ROS-mediated RhoA/Rho kinase pathway in rat hypertension induced by chronic intermittent hypoxia” [84]. Confirming other reports of increased Rhoa in PH [83], we found *Rhoa* as upregulated by 7.00× in HO, 6.78× in CM, and 9.21× in HM. Our results complete the results of a previous report [85] by showing that *Rhoa* is upregulated and plays an important role regardless of what caused the PH. Moreover, we found also that *Rhoa*’s action is mediated by the ATPases, a result that needs further investigation, especially in the light of the recent discovery of pathogenic mutations of the (not quantified in our study) ATP13A3 gene in IPAH [86].

**Figure 6:**
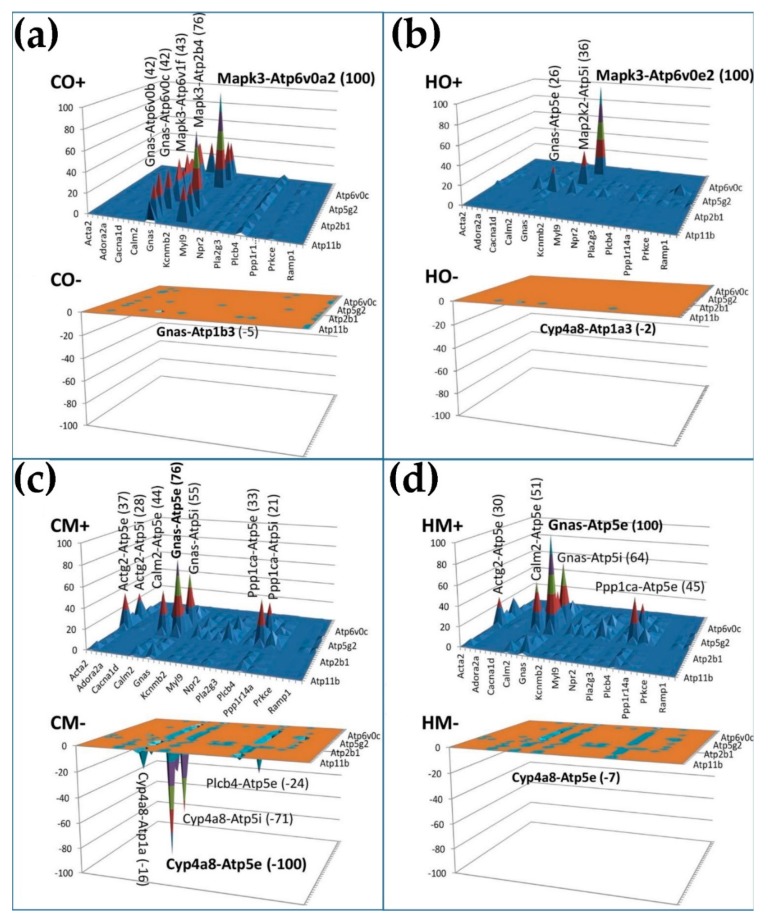
Gene pair prominence (GPP) analysis of the interaction of vascular smooth muscle contraction genes with the ATPases in the four conditions. (**a**) GPP values for the 472 positively (ρ > 0.9) and 36 negatively (ρ < −0.9) correlated gene pairs in CO; (**b**) GPP values for the 280 positively and four negatively correlated gene pairs in HO; (**c**) GPP values for the 455 positively and 115 negatively correlated gene-pairs in CM; (**d**) GPP values for the 262 positively and four negatively correlated gene-pairs in HM.

The gene pair prominence (GPP) analysis demonstrated the plasticity of the genomic fabrics and their interplay. Figure 6 illustrates how the GPP landscape of the interaction between the vascular smooth muscle contraction (VSMC) genes and the ATPases is remodeled in the three PH conditions with respect to control. Interestingly, while, in control, a few VSMC genes interact with almost all ATPases, in all three PH models, almost all VSMC genes interact with a few ATPases, making the vascular smooth muscle contraction more vulnerable to certain point alteration within the respiratory chain. This new type of analysis can be used to identify the most important gene pairs in specific interactions.

## 5. Conclusions

All PH groups exhibited abnormal expression of genes involved in inflammation, cell proliferation, and vascular smooth muscle contraction. Importantly, monocrotaline-treated rats that were exposed to hypoxia, which shows similar changes to PAH, displayed evidence of mitochondrial damage and activation of antioxidants, which are likely to cause metabolic switch and provide a survival milieu for the proliferating cells. Our finding of the reversed correlation of *Rhoa* with ATPases from negative in control rats to positive in PH suggests a potential therapeutic avenue by targeting *Rhoa* and/or its partners, as suggested in 2008 [87].

## Figures and Tables

**Table 1:** Regulation of the chemokine signaling pathway (Kyoto Encyclopedia of Genes and Genomes (KEGG), map rno04062) genes. Significant (larger than cut-off (CUT)) absolute fold-changes are in bold positive numbers for upregulated and bold negative numbers for downregulated. Note the range of the CUTs, from 1.24× (for the regulation of *Rhoa* in HM vs. CO) to 3.60× (for the regulation of *Nfkbia* in HO vs. CO). Our procedure to determine the cut-off for the absolute fold-change for every gene in each comparison instead of using a fixed cut-off (like 1.5×) identified additional regulated genes (, e.g., *Ccr1l1* in HO/CO). It also eliminated the false regulations (e.g., *Bcar1* in HO/CO), whose absolute (although over 1.5×) fold-change was below the CUT (2.98) computed for that gene in the compared conditions.

Gene	Description	HO/CO	CUT	CM/CO	CUT	HM/CO	CUT
*Adcy2*	Adenylate cyclase 2 (brain)	**−4.24**	2.32	**−4.72**	2.33	**−4.21**	2.34
*Akt1*	V-akt murine thymoma viral oncogene homolog 1	−1.42	1.61	1.09	1.94	**1.80**	1.78
*Arrb1*	Arrestin, β 1	**−9.55**	2.86	**−41.04**	2.39	**−52.82**	2.53
*Arrb2*	Arrestin, β 2	−1.38	2.11	**3.37**	2.54	**2.95**	2.24
*Bcar1*	Breast cancer anti-estrogen resistance 1	1.74	2.98	**5.27**	2.68	**10.39**	1.83
*Ccl21*	Chemokine (C-C motif) ligand 21	**3.26**	2.70	**79.95**	3.31	**247.07**	1.34
*Ccl24*	Chemokine (C-C motif) ligand 24	**5.07**	3.07	**10.24**	2.36	**5.74**	2.13
*Ccl27*	Chemokine (C-C motif) ligand 27	1.18	3.00	**−7.76**	1.85	**−10.14**	2.14
*Ccl5*	Chemokine (C-C motif) ligand 5	−1.35	1.85	**4.56**	2.45	**4.40**	1.65
*Ccl6*	C-C motif chemokine 6	**7.53**	3.32	**22.93**	2.72	**38.26**	1.98
*Ccl9*	Chemokine (C-C motif) ligand 9	−1.18	1.88	**3.75**	2.68	**2.16**	1.82
*Ccr1l1*	Chemokine (C-C motif) receptor 1-like 1	**−1.48**	1.46	−1.66	1.83	**−2.77**	1.80
*Ccr9*	Chemokine (C-C motif) receptor 9	**4.01**	2.84	**10.35**	2.65	1.29	1.78
*Cx3cr1*	Chemokine (C-X3-C motif) receptor 1	2.48	2.96	2.65	2.99	**−2.53**	2.35
*Cxcl12*	Chemokine (C-X-C motif) ligand 12	1.77	2.38	**3.77**	2.41	**60.14**	1.83
*Cxcl3*	Chemokine (C-X-C motif) ligand 3	1.61	2.33	**3.93**	3.13	**3.33**	1.93
*Gnai2*	Guanine nucleotide binding protein (G protein), α inhibiting 2	**4.30**	3.59	**5.79**	2.95	**10.05**	2.13
*Gnb1*	Guanine nucleotide binding protein (G protein), β polypeptide 1	**−4.22**	2.37	−1.47	2.80	1.23	2.19
*Gnb2*	Guanine nucleotide binding protein (G protein), β polypeptide 2	2.15	2.99	**3.44**	2.49	**6.23**	1.70
*Gnb3*	Guanine nucleotide binding protein (G protein), β polypeptide 3	**−3.33**	2.17	**−6.18**	2.40	**−11.78**	2.42
*Gng13*	Guanine nucleotide binding protein (G protein), γ 13	**−2.64**	2.43	**−3.56**	2.36	**−2.33**	2.10
*Gng3*	Guanine nucleotide binding protein (G protein), γ 3	**−22.18**	2.46	**−21.14**	2.51	**−32.06**	2.44
*Gng4*	guanine nucleotide binding protein (G protein), γ 4 subunit	−1.24	1.46	−1.29	1.77	**−2.19**	1.71
*Gngt2*	Guanine nucleotide binding protein (G protein), γ transducing activity polypeptide 2	1.76	2.62	**7.16**	2.75	**11.11**	1.63
*Grb2*	Growth factor receptor bound protein 2	**4.31**	2.79	**3.69**	2.75	**6.32**	1.87
*Grk6*	G protein-coupled receptor kinase 6	**−3.00**	2.59	−1.04	2.69	1.69	2.07
*Gsk3a*	Glycogen synthase kinase 3 α	**−4.67**	2.17	**−4.44**	2.31	**−7.27**	2.21
*Mapk3*	Mitogen activated protein kinase 3	1.72	2.92	**−3.71**	2.05	**−3.42**	1.89
*Nfkbia*	Nuclear factor of kappa light polypeptide gene enhancer in B-cells inhibitor, α	1.92	3.60	**5.56**	3.03	**9.00**	2.20
*Nfkbid*	NF-κB inhibitor delta	**−1.79**	1.64	**−2.08**	1.89	**−3.25**	1.85
*Pak1*	P21 protein (Cdc42/Rac)-activated kinase 1	−1.79	2.19	**−3.31**	2.17	**−3.17**	2.15
*Pik3r1*	Phosphoinositide-3-kinase, regulatory subunit 1 (α)	1.20	2.03	**2.77**	2.28	**4.25**	1.28
*Pik3r5*	Phosphoinositide-3-kinase, regulatory subunit 5	**−11.53**	2.66	**−5.87**	2.40	**−7.66**	2.38
*Plcb4*	Phospholipase C, β 4	**3.18**	2.86	**6.70**	2.99	**−4.79**	2.12
*Ppbp*	Pro-platelet basic protein (chemokine (C-X-C motif) ligand 7)	−1.05	1.41	−1.16	2.11	**−3.06**	2.13
*Ptk2b*	PTK2B protein tyrosine kinase 2 β	**−12.47**	2.45	**−6.89**	2.36	**−8.51**	2.34
*Rap1b*	RAP1B, member of RAS oncogene family	**−3.04**	2.84	−1.84	3.17	−1.17	2.37
*Rela*	V-rel reticuloendotheliosis viral oncogene homolog A (avian)	**−3.38**	2.07	−1.15	2.29	1.22	1.98
*Rhoa*	Ras homolog gene family, member A	**7.00**	2.80	**6.78**	2.52	**9.21**	1.24
*Shc1*	SHC (Src homology 2 domain containing) transforming protein 1	**−2.71**	2.09	**−4.11**	2.03	**−4.94**	2.08
*Sos1*	Son of sevenless homolog 1 (Drosophila)	1.02	1.99	2.07	2.49	**−2.86**	1.77
*Tiam1*	T-cell lymphoma invasion and metastasis 1	**−2.79**	1.75	**−2.67**	2.13	**−4.85**	1.95
*Vav2*	Vav 2 guanine nucleotide exchange factor	2.06	2.74	**3.44**	2.43	**6.02**	1.45
*Xcl1*	Chemokine (C motif) ligand 1	−1.51	1.96	−1.53	2.20	**−3.49**	2.03

**Table 2:** Significantly up- (positive fold-change in bold numbers) and downregulated (negative fold-change in bold numbers) ATPases, cytochrome c oxidases, and NADH dehydrogenases in the three PH rat models. Note the range of the CUTs, from 1.24 (for the regulation of *Ndufv3* in HM vs. CO) to 3.68 (for the regulation of *Cox6a1* in HO vs. CO). Our procedure to determine the cut-off for the absolute fold-change for every gene in each comparison instead of using a fixed cut-off (like 1.5×) eliminated the false regulations (expression ratio in Italics, e.g., *Atp11b* in HO/CO), whose absolute (although over 1.5×) fold-change was below the CUT (2.28) computed for that gene in the compared conditions.

Gene	Description	HO/CO	CUT	CM/CO	CUT	HM/CO	CUT
*Atp11b*	ATPase phospholipid transporting 11B	*−1.74*	2.28	*1.34*	2.46	**2.05**	1.82
*Atp13a2*	ATPase type 13A2	*−1.67*	2.18	*1.55*	2.40	**2.11**	2.00
*Atp1a1*	ATPase, Na+/K+ transporting, α 1 polypeptide	*−1.78*	3.34	**4.01**	3.08	**6.65**	2.25
*Atp1a3*	ATPase, Na+/K+ transporting, α 3 polypeptide	*−1.52*	1.71	*−1.86*	1.95	**−3.48**	2.07
*Atp1b2*	ATPase, Na+/K+ transporting, β 2 polypeptide	**−146.48**	3.21	**−257.37**	2.51	**−111.20**	2.47
*Atp1b3*	ATPase, Na+/K+ transporting, β 3 polypeptide	*2.14*	2.32	**12.00**	2.35	**15.88**	1.26
*Atp2a2*	ATPase, Ca++ transporting, cardiac muscle, slow twitch 2	*1.56*	2.48	**4.83**	2.46	**10.66**	1.49
*Atp2a3*	ATPase, Ca++ transporting, ubiquitous	*1.40*	1.95	*1.62*	1.87	**1.88**	1.39
*Atp2b1*	ATPase, Ca++ transporting, plasma membrane 1	**−3.70**	3.00	*−1.19*	2.86	*1.30*	2.34
*Atp2b4*	ATPase, Ca++ transporting, plasma membrane 4	**−2.48**	1.90	*−1.69*	2.59	**−11.70**	2.28
*Atp2c1*	ATPase, Ca++ transporting, type 2C, member 1	*−1.35*	1.67	*1.24*	1.69	**1.85**	1.50
*Atp4a*	ATPase, H+/K+ exchanging, α polypeptide	*1.13*	1.59	*−1.12*	1.94	**−2.35**	1.61
*Atp5d*	ATP synthase, H+ transporting, mitochondrial F1 complex, delta subunit	*2.27*	3.21	**6.84**	2.82	**9.15**	1.92
*Atp5f1*	ATP synthase, H+ transporting, mitochondrial Fo complex, subunit B1	**4.36**	3.01	**27.45**	2.92	**51.30**	1.67
*Atp5g1*	ATP synthase, H+ transporting, mitochondrial Fo complex, subunit C1	**4.71**	3.18	**10.84**	2.78	**16.08**	2.08
*Atp5g2*	ATP synthase, H+ transporting, mitochondrial Fo complex, subunit C2	**3.21**	2.91	**14.44**	2.84	**26.97**	1.86
*Atp5g3*	ATP synthase membrane subunit c locus 3	**−7.56**	3.23	*−1.08*	3.22	*1.35*	2.35
*Atp5i*	ATP synthase, H+ transporting, mitochondrial Fo complex, subunit E	**17.18**	2.97	**11.45**	2.89	**15.65**	2.19
*Atp5j2*	ATP synthase, H+ transporting, mitochondrial Fo complex, subunit F2	−1.27	2.91	**5.30**	2.90	**8.37**	2.24
*Atp6v0a1*	ATPase, H+ transporting, lysosomal V0 subunit A1	**4.14**	2.57	**3.11**	2.35	**4.83**	1.65
*Atp6v0a2*	ATPase, H+ transporting, lysosomal V0 subunit A2	**−2.76**	2.21	*−2.35*	2.37	*−1.52*	2.16
*Atp6v0b*	ATPase, H+ transporting, lysosomal V0 subunit B	**−6.71**	3.10	*−1.45*	3.18	*−1.31*	2.59
*Atp6v0c*	ATPase, H+ transporting, lysosomal V0 subunit C	**−31.61**	2.78	**−5.37**	3.13	**−3.65**	2.39
*Atp6v0e1*	ATPase, H+ transporting, lysosomal, V0 subunit e1	*1.78*	2.25	**16.44**	2.87	**26.14**	1.32
*Atp6v0e2*	ATPase, H+ transporting V0 subunit e2	*1.84*	2.59	**−3.40**	1.79	**−5.62**	1.95
*Atp6v1f*	ATPase, H transporting, lysosomal V1 subunit F	*1.10*	2.22	**5.42**	2.78	**9.44**	1.53
*Atp6v1g2*	ATPase, H+ transporting, lysosomal V1 subunit G2	**−11.80**	2.62	**−16.92**	2.68	**−39.54**	2.66
*Atp7b*	ATPase, Cu++ transporting, β polypeptide	*−1.10*	1.95	*−1.61*	2.13	**−4.23**	1.93
*Atpif1*	ATPase inhibitory factor 1	*1.06*	3.10	**5.02**	3.13	**8.16**	2.21
*Cox14*	cytochrome c oxidase assembly factor COX14	**−3.02**	2.38	*1.25*	2.48	*1.45*	2.12
*Cox17*	cytochrome c oxidase assembly homolog 17	*−1.43*	3.42	*2.09*	3.07	**3.01**	2.28
*Cox18*	cytochrome c oxidase assembly homolog 18	**−2.41**	2.11	*−1.51*	2.20	*−1.23*	2.03
*Cox4i1*	cytochrome c oxidase subunit IV isoform 1	**11.99**	2.91	**30.01**	2.81	**52.39**	1.89
*Cox4i2*	cytochrome c oxidase subunit IV isoform 2 (lung)	**−6.30**	3.02	**−4.07**	2.77	**−2.89**	2.32
*Cox5b*	cytochrome c oxidase subunit Vb	**−9.14**	2.93	*−1.85*	3.15	*−1.02*	2.34
*Cox6a1*	cytochrome c oxidase, subunit VIa, polypeptide 1	*−1.85*	3.68	*1.66*	3.10	**2.87**	2.24
*Cox6a2*	cytochrome c oxidase subunit VIa polypeptide 2	*1.33*	2.05	**2.99**	2.60	**16.89**	2.29
*Cox6b1*	cytochrome c oxidase subunit 6B1	**4.27**	2.82	**−3.59**	2.54	**−3.02**	2.29
*Cox6b2*	cytochrome c oxidase subunit VIb polypeptide 2	**−3.95**	2.64	**−7.82**	2.34	**−7.12**	2.32
*Cox6c*	cytochrome c oxidase, subunit VIc	**4.56**	3.49	**11.35**	2.92	**18.57**	2.02
*Cox7a2*	cytochrome c oxidase subunit VIIa polypeptide 2	*1.85*	2.97	**7.68**	2.74	**12.31**	1.95
*Cox7b*	cytochrome c oxidase subunit VIIb	*1.50*	2.03	**6.58**	2.77	**11.58**	1.79
*Cox8a*	cytochrome c oxidase subunit VIIIa	−4.07	3.07	*1.27*	3.18	**2.50**	2.33
*Cox8b*	cytochrome c oxidase, subunit VIIIb	**2.96**	2.01	**2.35**	1.93	**6.01**	2.37
*Coa5*	cytochrome C oxidase assembly factor 5	−20.24	2.92	−8.03	3.05	−11.72	2.93
*Ndufa10*	NADH dehydrogenase (ubiquinone) 1 α subcomplex 10	*1.24*	2.99	*1.81*	2.56	**3.27**	1.92
*Ndufa11*	NADH dehydrogenase (ubiquinone) 1 α subcomplex 11	*−1.02*	2.24	**3.51**	2.08	**3.92**	1.72
*Ndufa13*	NADH:ubiquinone oxidoreductase subunit A13	*−2.20*	3.07	*3.05*	3.10	**5.35**	2.18
*Ndufa3*	NADH dehydrogenase (ubiquinone) 1 α subcomplex, 3	**−4.15**	2.81	**−2.79**	2.51	*−2.08*	2.25
*Ndufa6*	NADH dehydrogenase (ubiquinone) 1 α subcomplex, 6 (B14)	**4.42**	3.33	**15.50**	2.84	**28.28**	1.71
*Ndufaf5*	NADH dehydrogenase (ubiquinone) complex I, assembly factor 5	*−1.16*	2.21	*1.50*	2.45	**2.21**	1.94
*Ndufb10*	NADH dehydrogenase (ubiquinone) 1 β subcomplex, 10	*2.67*	3.13	**4.78**	2.67	**7.57**	1.63
*Ndufb2*	NADH dehydrogenase (ubiquinone) 1 β subcomplex, 2	**7.38**	3.22	**20.76**	2.78	**35.55**	1.73
*Ndufb3*	NADH dehydrogenase (ubiquinone) 1 β subcomplex 3	**3.15**	2.92	**7.29**	2.42	**12.14**	1.60
*Ndufb4*	NADH dehydrogenase (ubiquinone) 1 β subcomplex 4	**3.52**	3.35	**11.33**	2.85	**21.34**	1.89
*Ndufb5*	NADH dehydrogenase (ubiquinone) 1 β subcomplex, 5	*1.31*	2.83	**4.31**	2.77	**6.50**	1.94
*Ndufb6*	NADH dehydrogenase (ubiquinone) 1 β subcomplex, 6	**−6.75**	2.75	*−1.20*	3.14	*1.33*	2.28
*Ndufb7*	NADH dehydrogenase (ubiquinone) 1 β subcomplex, 7	**−4.28**	3.63	*−1.59*	3.12	*−1.12*	2.32
*Ndufb8*	NADH dehydrogenase (ubiquinone) 1 β subcomplex 8	*2.64*	3.37	**7.96**	3.09	**13.61**	2.35
*Ndufb9*	NADH dehydrogenase (ubiquinone) 1 β subcomplex, 9	*1.67*	2.89	**11.81**	2.95	**19.58**	2.16
*Ndufs3*	NADH dehydrogenase (ubiquinone) Fe-S protein 3	**−5.54**	3.14	*−1.39*	3.14	*1.08*	2.42
*Ndufs5*	NADH dehydrogenase (ubiquinone) Fe-S protein 5	**3.54**	2.87	**12.91**	2.84	**23.34**	1.94
*Ndufs7*	NADH dehydrogenase (ubiquinone) Fe-S protein 7	**5.04**	3.35	**8.90**	2.68	**10.68**	1.74
*Ndufv1*	NADH dehydrogenase (ubiquinone) flavoprotein 1	**5.55**	3.38	**8.51**	2.82	**14.54**	1.94
*Ndufv3*	NADH dehydrogenase (ubiquinone) flavoprotein 3	*1.32*	1.95	*2.12*	2.22	**3.71**	1.24
*Sdhaf2*	succinate dehydrogenase complex assembly factor 2	*2.15*	2.69	**4.59**	2.76	**7.66**	1.73

## Supplementary Materials

The following are available online at https://www.mdpi.com/2073-4425/11/2/126/s1: Table S1: Regulation of important genes involved in the immune-inflammatory response; Table S2: Regulation of mitochondrial genes.
