# Supplementary material for: Pulmonary Hypertension Remodels the Genomic Fabrics of Major Functional Pathways
Source: Genes (Basel). 2020 Jan 23;11(2):126. doi: 10.3390/genes11020126 (PMC7074533; doi:10.3390/genes11020126)
Supplement: Supplementary file 1 [file genes-11-00126-s001.pdf]

Supplementary Materials

Table S1. Regulation of important genes involved in the immune-inflammatory response. Significant (larger than CUT) fold-changes are in positive bold numbers for upregulated and negative bold numbers for downregulated. Note the range of the CUTs, from 1.12 (for the regulation of *Cxcr7* in CM vs. CO) to 3.33 (for the regulation of *Il34* in both HO and CM vs. CO). Our procedure to determine the cut-off for the absolute fold-change for every gene in each comparison instead of using a fixed cut-off (like 1.5×) identified additional regulated genes (CUT in yellow background, e.g., *Ccl7* in HO/CO). It also eliminated the false regulations (expression ratio in gray background, e.g., *Cxcl16* in HO/CO), whose absolute (although over 1.5×) fold-change was below the CUT (2.57) computed for that gene in the compared conditions.

Gene	Description	HO/CO	CUT	CM/CO	CUT	HM/CO	CUT
Ccl11	chemokine (C-C motif) ligand 11	*1.19*	1.95	*1.36*	1.95	**3.56**	1.72
Ccl17	chemokine (C-C motif) ligand 17	*-1.10*	1.76	**2.21**	1.76	**1.49**	1.39
Ccl19	chemokine (C-C motif) ligand 19	**2.84**	2.59	**7.88**	2.59	**17.82**	2.72
Ccl2	chemokine (C-C motif) ligand 2	**2.18**	2.18	**3.58**	2.18	**5.12**	1.74
Ccl21	chemokine (C-C motif) ligand 21	**3.26**	2.70	**79.95**	2.70	**247.07**	1.34
Ccl24	chemokine (C-C motif) ligand 24	**5.07**	3.07	**10.24**	3.07	**5.74**	2.13
Ccl3	chemokine (C-C motif) ligand 3	**1.41**	1.13	**2.29**	1.13	**2.76**	1.45
Ccl5	chemokine (C-C motif) ligand 5	*-1.35*	1.85	**4.56**	1.85	**4.40**	1.65
Ccl6	chemokine (C-C motif) ligand 6	**4.04**	2.83	**54.71**	2.83	**74.73**	1.80
Ccl6	C-C motif chemokine 6	**7.53**	3.32	**22.93**	3.32	**38.26**	1.98
Ccl7	chemokine (C-C motif) ligand 7	*-1.01*	1.39	**1.52**	1.39	**1.95**	1.74
Ccl9	chemokine (C-C motif) ligand 9	*-1.18*	1.88	**3.75**	1.88	**2.16**	1.82
Ccr9	chemokine (C-C motif) receptor 9	**4.01**	2.84	**10.35**	2.84	*1.29*	1.78
Ciapin1	cytokine induced apoptosis inhibitor 1	*1.40*	2.08	**3.16**	2.08	**5.16**	1.35
Crlf1	cytokine receptor-like factor 1	**4.15**	3.32	**10.68**	3.32	**12.63**	1.53
Crlf2	cytokine receptor-like factor 2	*1.38*	3.04	*2.91*	3.04	**4.13**	1.99
Crlf3	cytokine receptor-like factor 3	**1.30**	1.25	**2.09**	1.25	**2.54**	1.55
Cxcl1	chemokine (C-X-C motif) ligand 1 (melanoma growth stimulating activity, alpha)	**1.52**	1.20	**4.48**	1.20	**5.56**	1.71
Cxcl12	chemokine (C-X-C motif) ligand 12	*1.77*	2.38	**3.77**	2.38	**60.14**	1.83
Cxcl14	chemokine (C-X-C motif) ligand 14	**6.99**	2.67	**15.02**	2.67	**15.57**	1.69
Cxcl16	chemokine (C-X-C motif) ligand 16	*2.21*	2.57	**11.83**	2.57	**15.54**	1.28
Cxcl17	chemokine (C-X-C motif) ligand 17	**9.78**	2.87	**26.72**	2.87	**27.68**	1.55
Cxcl2	chemokine (C-X-C motif) ligand 2	*1.00*	1.22	**1.96**	1.22	*1.10*	2.03
Cxcl3	chemokine (C-X-C motif) ligand 3	*1.61*	2.33	**3.93**	2.33	**3.33**	1.93
Cxcr1	chemokine (C-X-C motif) receptor 1	*1.24*	1.26	**1.93**	1.26	**2.25**	1.66
Cxcr4	chemokine (C-X-C motif) receptor 4	*-1.21*	2.38	**4.56**	2.38	**2.84**	1.78

Cxcr7	chemokine (C-X-C motif) receptor 7	*-1.02*	1.12	**1.35**	1.12	**2.33**	1.64
Cytl1	cytokine like 1 (Cytl1)	*1.14*	1.25	**1.26**	1.25	*-1.56*	1.75
Ifi27	interferon, alpha-inducible protein 27	**35.46**	2.77	**57.06**	3.05	**57.04**	2.09
Ifi27l2b	interferon, alpha-inducible protein 27 like 2B	**8.92**	2.60	**39.94**	3.32	**62.37**	2.19
Ifi30	interferon gamma inducible protein 30	**4.83**	3.19	**42.25**	2.53	**49.68**	1.33
Ifi35	interferon-induced protein 35	*-1.08*	1.55	*1.94*	1.96	**2.65**	1.94
Ifi47	interferon gamma inducible protein 47	*1.05*	1.09	*1.50*	1.95	**1.29**	1.25
Ifit2	interferon-induced protein with tetratricopeptide repeats 2	*1.13*	1.41	*1.76*	2.07	**2.32**	1.55
Ifit3	interferon-induced protein with tetratricopeptide repeats 3	*1.01*	1.37	*2.29*	2.40	**2.25**	1.78
Ifitm1	interferon induced transmembrane protein 1	*1.28*	2.22	**10.29**	3.08	**20.26**	1.81
Ifitm2	interferon induced transmembrane protein 2	*-1.15*	3.14	**19.39**	3.15	**36.04**	2.22
Ifitm3	interferon induced transmembrane protein 3	*-1.16*	2.47	**9.57**	2.75	**19.11**	1.81
Ifngr1	interferon gamma receptor 1	*1.50*	3.28	**17.75**	2.54	**16.54**	1.85
Ifrd1	interferon-related developmental regulator 1	*1.08*	1.37	**2.90**	1.97	**3.26**	1.80
Ifrd2	interferon-related developmental regulator 2	*1.29*	1.45	**1.92**	1.75	**2.24**	1.41
Ik	IK cytokine	*1.73*	2.11	**3.60**	2.11	**4.52**	1.36
Il10rb	interleukin 10 receptor, beta	*-1.08*	2.94	*2.28*	2.94	**3.88**	1.84
Il12b	interleukin 12B	**1.54**	1.47	*-1.06*	1.47	*-1.94*	2.40
Il16	interleukin 16	*1.38*	1.92	**3.07**	1.92	**5.52**	1.32
Il17ra	interleukin 17 receptor A	**3.00**	2.65	**7.66**	2.65	**14.06**	1.40
Il17re	interleukin 17 receptor E	*1.33*	2.50	**2.71**	2.50	**4.34**	1.25
Il18	interleukin 18	**1.39**	1.37	**5.78**	1.37	**5.86**	1.53
Il1a	interleukin 1 alpha	*1.01*	1.61	**1.83**	1.61	*1.50*	1.62
Il1r2	interleukin 1 receptor, type II	**1.49**	1.09	**4.70**	1.09	**2.44**	1.74
Il1rn	interleukin 1 receptor antagonist	*-1.30*	1.85	**1.89**	1.85	**2.55**	1.74
Il34	interleukin 34	**5.71**	3.33	**18.05**	3.33	**27.98**	1.40
Il6st	interleukin 6 signal transducer	**2.75**	2.16	**7.87**	2.16	**13.02**	1.46
Ilf3	interleukin enhancer binding factor 3	*-1.17*	1.55	*1.26*	1.55	**1.51**	1.50
Tnfaip8	tumor necrosis factor, alpha-induced protein 8	**3.70**	2.74	**5.19**	2.23	**6.99**	1.40
Tnfaip8l2	tumor necrosis factor, alpha-induced protein 8-like 2	**4.39**	2.62	**8.29**	2.64	**20.10**	1.60
Tnfrsf11a	tumor necrosis factor receptor superfamily, member 11a, NFKB activator	**1.83**	1.73	*-1.51*	2.12	**-1.98**	1.73
Tnfrsf12a	tumor necrosis factor receptor superfamily, member 12a	*-1.64*	1.67	**3.16**	2.80	**3.98**	1.67
Tnfrsf14	tumor necrosis factor receptor superfamily, member 14	*1.04*	1.20	**1.48**	1.43	*1.38*	1.47
Tnfrsf1a	tumor necrosis factor receptor superfamily, member 1a	**5.32**	2.70	**6.12**	2.71	**12.67**	1.45
Tnfrsf21	tumor necrosis factor receptor superfamily, member 21	*1.57*	1.74	**3.96**	2.49	**2.17**	1.46
Tnfrsf26	tumor necrosis factor receptor superfamily, member 26	*1.56*	1.72	**1.97**	1.75	**2.82**	1.52
Tnfsf13	tumor necrosis factor (ligand) superfamily, member 13	*1.13*	2.32	**6.87**	2.43	**5.98**	1.64

Table S2. Regulation of mitochondrial genes. Significant (larger than CUT) absolute fold-changes are in positive bold numbers for upregulated and negative bold numbers for downregulated. Note the range of the CUTs, from 1.53 (for the regulation of *Mrpl10* in CM vs. CO) to 3.48 (for the regulation of *Mrpl28* in HO vs. CO). Our procedure to determine the cut-off for the absolute fold-change for every gene in each comparison instead of using a fixed cut-off (like 1.5×) eliminated the false regulations (expression ratio in gray background, e.g., *Mrpl13* in HO/CO), whose absolute (although over 1.5×) fold-change was below the CUT (2.58) computed for that gene in the compared conditions.

Gene	Description	HO/CO	CUT	CM/CO	CUT	HM/CO	CUT
Mrpl10	mitochondrial ribosomal protein L10	**18.83**	3.01	**13.96**	1.53	**16.23**	1.54
Mrpl13	mitochondrial ribosomal protein L13	*2.18*	2.58	**7.52**	2.58	**13.92**	1.76
Mrpl14	mitochondrial ribosomal protein L14	*2.41*	3.29	*1.57*	3.01	*2.32*	2.38
Mrpl17	mitochondrial ribosomal protein L17	**3.56**	3.04	**14.88**	2.76	**25.82**	1.66
Mrpl19	mitochondrial ribosomal protein L19	*1.82*	1.82	*1.17*	1.96	**1.76**	1.59
Mrpl2	mitochondrial ribosomal protein L2	*-1.70*	2.77	*-1.02*	2.64	*1.40*	2.11
Mrpl20	mitochondrial ribosomal protein L20	*-1.25*	3.28	*2.07*	3.15	**3.22**	2.30
Mrpl23	mitochondrial ribosomal protein L23	**-3.37**	2.99	*-1.96*	2.95	*-1.42*	2.24
mrpl24	mitochondrial ribosomal protein L24	*-1.00*	2.29	*1.87*	2.60	**2.90**	2.07
Mrpl27	mitochondrial ribosomal protein L27	*2.27*	3.16	**4.80**	2.82	**6.80**	1.92
Mrpl28	mitochondrial ribosomal protein L28	*3.22*	3.48	**4.11**	3.07	**6.62**	2.46
Mrpl34	mitochondrial ribosomal protein L34	*-2.41*	3.24	*1.28*	2.96	**2.20**	2.17
Mrpl35	mitochondrial ribosomal protein L35	**3.08**	2.13	**2.09**	1.87	**2.05**	1.70
Mrpl37	mitochondrial ribosomal protein L37	*-1.65*	3.10	*1.16*	2.92	*1.98*	2.15
Mrpl38	mitochondrial ribosomal protein L38	**-4.33**	2.46	**-3.15**	2.50	**-2.70**	2.10
Mrpl40	mitochondrial ribosomal protein L40	**-2.54**	2.05	**-2.31**	2.06	*-1.66*	1.87
Mrpl41	mitochondrial ribosomal protein L41	*1.79*	2.49	**6.10**	2.59	**10.69**	1.69
Mrpl42	mitochondrial ribosomal protein L42	**-3.32**	2.02	*-1.96*	2.32	*-1.44*	2.00
Mrpl45	mitochondrial ribosomal protein L45	*2.39*	2.61	**5.24**	2.66	**8.00**	1.61
Mrpl48	mitochondrial ribosomal protein L48	**-6.50**	3.23	**-3.66**	2.90	*-2.21*	2.48
Mrpl54	mitochondrial ribosomal protein L54	*-1.10*	3.32	**3.80**	3.16	**5.78**	2.29
Mrpl9	mitochondrial ribosomal protein L9	**3.14**	2.93	**6.66**	2.61	**13.75**	1.81
Mrps12	mitochondrial ribosomal protein S12	*2.13*	2.86	**4.22**	2.66	**5.81**	1.80
Mrps15	mitochondrial ribosomal protein S15	*1.24*	1.91	*1.59*	1.68	*1.57*	1.77
Mrps2	mitochondrial ribosomal protein S2	**-4.43**	2.19	**-3.83**	2.27	**-5.21**	2.17
Mrps21	mitochondrial ribosomal protein S21	**-2.74**	2.18	**-2.45**	2.15	**-3.18**	2.28
